# Confirmation of the Need for Reclassification of *Neisseria mucosa* and *Neisseria sicca* Using Average Nucleotide Identity Blast and Phylogenetic Analysis of Whole-Genome Sequencing: Hinted by Clinical Misclassification of a *Neisseria mucosa* Strain

**DOI:** 10.3389/fmicb.2021.780183

**Published:** 2022-02-21

**Authors:** Yanqi Jin, Hao Xu, Qing Yao, Beiqing Gu, Zhouhan Wang, Tianyuan Wang, Xiaopeng Yu, Yingfeng Lu, Beiwen Zheng, Yimin Zhang

**Affiliations:** ^1^State Key Laboratory for Diagnosis and Treatment of Infectious Diseases, National Clinical Research Center for Infectious Diseases, Collaborative Innovation Center for Diagnosis and Treatment of Infectious Diseases, The First Affiliated Hospital, College of Medicine, Zhejiang University, Hangzhou, China; ^2^Department of Infectious Diseases, Haining People’s Hospital, Haining, China; ^3^Department of Clinical Laboratory, Haining People’s Hospital, Haining, China

**Keywords:** *Neisseria mucosa*, *Neisseria sicca*, *Neisseria subflava*, reclassification, whole-genome sequencing, average nucleotide identity blast, phylogenetic analysis

## Abstract

The taxonomy of the genus *Neisseria* remains confusing, particularly regarding *Neisseria mucosa* and *Neisseria sicca.* In 2012, ribosomal multi-locus sequence typing reclassified both as *N. mucosa*, but data concerning 17 *N. sicca* strains remain available in GenBank. The continuous progress of high-throughput sequencing has facilitated ready accessibility of whole-genome data, promoting vigorous development of average nucleotide identity (ANI) and high-resolution phylogenetic analysis. Here, we report that a *Neisseria* isolate, which caused native-valve endocarditis and multiple embolic brain infarcts in a patient with congenital heart disease, was misidentified as *N. sicca* by VITEK MS. This isolate was reclassified as *N. mucosa* by ANI blast (ANIb) and by phylogenetic analysis using whole-genome data yielded by the PacBio Sequel and Illumina NovaSeq PE150 platforms. The confusion evident in the GenBank and matrix-assisted laser desorption/ionization time-of-flight mass spectrometry (MALDI-TOF MS) databases suggests that *N. mucosa* (*n* = 13) and *N. sicca* (*n* = 16) in GenBank should be reclassified using ANIb and high-resolution phylogenetic analysis. The whole-genome data of 30 strains (including the clinical isolate) were compared with the data of 27 type *Neisseria* strains (including one *N. sicca* and two *N. mucosa* type strains) as a genomic index. In total, 25 (8 originally identified as *N. mucosa* and 17 originally identified as *N. sicca*) and 7 (1 originally identified as *N. sicca* and 6 originally identified as *N. mucosa*) strains were reclassified into the *N. mucosa* and *Neisseria subflava* groups, respectively; 1 residual *N. mucosa* strain was reclassified as *Neisseria meningitidis*. In conclusion, a combination of ANIb and robust phylogenetic analysis reclassified strains originally identified as *N. mucosa* and *N. sicca* into (principally) the *N. mucosa* group and the *N. subflava* group. The misclassified *N. sicca* and *N. mucosa* strains in the GenBank and MALDI-TOF MS databases were supposed to be corrected. Updated genomic classification strategy for originally identified *N. mucosa* and *N. sicca* strains was recommended to be adopted in GenBank.

## Introduction

*Neisseria mucosa* and *Neisseria sicca* are both non-pathogenic species that inhabit the human pharynx (Caugant and Brynildsrud, 2020). However, they are phenotypically similar; *N. mucosa* forms mucoid colonies and reduces nitrates, whereas *N. sicca* forms dry, wrinkled colonies and does not reduce nitrates (Tønjum, 2005). Both species exhibit variable features; identification to the species level using phenotypic characters is difficult. Compared with traditional phenotypic approaches, genomic analyses are superior (Paul et al., 2019). Whole-genome sequencing (WGS) is now very accessible and affordable (Zong, 2020). On the basis of ribosomal multi-locus sequence typing (rMLST) findings, Bennett et al. (2012) proposed that *N. sicca* was a variant of *N. mucosa*. Using a phylogenetic taxonomic method, Caugant and Brynildsrud (2020) suggested that *N. sicca* and *N. mucosa* did not form monophyletic groups and might constitute a single species. Although the *N. sicca* strains evaluated by Bennett et al. (2012) have been corrected in GenBank, a couple of *N. sicca* strains remain.

Current, WGS-based, high-resolution, genomic taxonomic methods include the average nucleotide identity blast (ANIb) and phylogenetic analysis. ANIb of all genes conserved in two genomes measures the evolutionary distance between the strains (Altschul et al., 1997; Konstantinidis and Tiedje, 2005). The phylogenetic analysis uses gene sequences to infer the evolutionary pattern (Staley, 2006). Both algorithms are always used together; they are complementary. For example, of 181 *Neisseria* isolates examined, seven putative novel *Neisseria* species were identified by ANI and phylogenetic analysis (Diallo et al., 2019).

Here, we report the case of the clinical isolate SAMN18451419, which was identified as *N. sicca* by VITEK MS; it was reclassified as *N. mucosa* by ANIb and phylogenetic analysis (*via* WGS using the PacBio Sequel and Illumina NovaSeq PE150 platforms). This *Neisseria* strain was isolated from a patient with infective native-valve endocarditis and multiple embolic brain infarcts. This case and the findings by Bennett et al. (2012) and Caugant and Brynildsrud (2020) suggest that *N. sicca* and *N. mucosa* might be the same species. We thus used phylogenetic and ANIb analysis to reassign the “*N. mucosa*” and “*N. sicca*” strains in GenBank to the correct groups.

## Materials and Methods

### Ethics Approval Statement

The study was conducted in accordance with the Declaration of Helsinki and was approved by the Clinical Research Ethics Committee of the First Affiliated Hospital, Zhejiang University School of Medicine [approval no. 2021IIT 026 (fast track)].

### Public Sequence Data

Whole-genome data from 56 strains were downloaded from GenBank using ‘‘*Neisseria*’’ as the search term^1^. Of these 56 strains, we selected 27 available from the American Type Culture Collection (ATCC) and National Collection of Type Cultures (NCTC). We downloaded all genome data of *Neisseria meningitidis* (*n* = 2), *Neisseria gonorrhoeae* (*n* = 2), *Neisseria elongata* (*n* = 2), *Neisseria lactamica* (*n* = 2), *N. mucosa* (*n* = 2), *N. sicca* (*n* = 1), *Neisseria weaveri* (*n* = 2), *Neisseria cinerea* (*n* = 2), *Neisseria flavescens* (*n* = 2), *Neisseria animalis* (*n* = 2), *Neisseria canis* (*n* = 2), *Neisseria polysaccharea* (*n* = 1), *Neisseria subflava* (*n* = 1), *Neisseria bacilliformis* (*n* = 1), *Neisseria animaloris* (*n* = 1), *Neisseria dentiae* (*n* = 1), *Neisseria zoodegmatis* (*n* = 1), and previously identified *N. mucosa* (*n* = 13) and *N. sicca* (*n* = 16) (Table 1).

**TABLE 1:** Information regarding 57 *Neisseria* strains used in this study.

No.	Species	Strain	GenBank strain BioSample	Assembly accession	Level	Release date
1	*Neisseria mucosa*	DRR241310-mag-bin.1	SAMEA8391525	GCA_905370285.1	Contig	2021-03-25
2	*Neisseria mucosa*	N32	SAMEA5208239	GCA_900654175.1	Contig	2019-09-19
3	*Neisseria mucosa*	*Neisseria meningitidis*	SAMEA104189262	GCA_900201385.1	Contig	2017-11-22
4	*Neisseria mucosa*	ATCC 25996	SAMN00008841	GCA_000173875.1	Contig	2009-02-04
5	*Neisseria mucosa*	C102	SAMN02463751	GCA_000186165.1	Scaffold	2011-01-11
6	*Neisseria mucosa*	C6A	SAMN02996611	GCA_000763635.1	Contig	2014-10-06
7	*Neisseria mucosa*	CCH7-A10	SAMN04299442	GCA_001556335.1	Contig	2016-02-10
8	*Neisseria mucosa*	FDAARGOS_260	SAMN04875586	GCA_002073715.2	Complete	2017-03-29
9	*Neisseria mucosa*	C2004002444	SAMN08299197	GCA_003044395.1	Contig	2018-04-09
10	*Neisseria mucosa*	C2008000159	SAMN08299202	GCA_003044445.1	Contig	2018-04-09
11	*Neisseria mucosa*	FDAARGOS_758	SAMN11056473	GCA_013267835.1	Complete	2020-06-04
12	*Neisseria mucosa*	L3_114_237G1_dasL3_114_237G1_concoct_13	SAMN17800961	GCA_018364365.1	Scaffold	2021-05-16
13	*Neisseria mucosa*	L2_013_000G1_dasL2_013_000G1_maxbin2.maxbin.018	SAMN17801510	GCA_018373375.1	Contig	2021-05-16
14	*Neisseria sicca*	VK64	SAMN00761806	GCA_000260655.1	Contig	2012-04-30
15	*Neisseria sicca*	Neisseria_sicca_BgEED21	SAMEA5664365	GCA_901873385.1	Contig	2021-02-13
16	*Neisseria sicca*	4320	SAMN02471879	GCA_000193735.2	Contig	2010-12-29
17	*Neisseria sicca*	DS1	SAMN02471880	GCA_000193755.2	Contig	2010-12-29
18	*Neisseria sicca*	UMB0321	SAMN08193710	GCA_002863285.1	Scaffold	2017-12-28
19	*Neisseria sicca*	C2007003584	SAMN08299201	GCA_003044425.1	Contig	2018-04-09
20	*Neisseria sicca*	C2010005502	SAMN08299211	GCA_003044565.1	Contig	2018-04-09
21	*Neisseria sicca*	C2014002478	SAMN08299228	GCA_003044765.1	Contig	2018-04-09
22	*Neisseria sicca*	DE0367	SAMN11792527	GCA_007673275.1	Scaffold	2019-07-30
23	*Neisseria sicca*	DE0493	SAMN11792653	GCA_007667125.1	Scaffold	2019-07-30
24	*Neisseria sicca*	DE0496	SAMN11792656	GCA_007667085.1	Scaffold	2019-07-30
25	*Neisseria sicca*	JCVI_32_bin.62	SAMN14570845	GCA_015263535.1	Scaffold	2020-11-06
26	*Neisseria sicca*	JCVI_39_bin.1	SAMN14570846	GCA_015263505.1	Scaffold	2020-11-06
27	*Neisseria sicca*	DSM 17713	SAMN15603184	GCA_014054945.1	Complete	2020-08-04
28	*Neisseria sicca*	LPB0402	SAMN20114574	GCA_019334765.1	Complete	2021-07-25
29	*Neisseria sicca*	C2006001571	SAMN08299199	GCA_003044345.1	Contig	2018-04-09
30	*Neisseria sicca*	NS20201025	SAMN18451419	GCA_017753665.1	Complete	2021-04-05
31	*Neisseria meningitidis*	NCTC3372	SAMEA104307687	GCA_900638575.1	Complete	2018-12-19
32	*Neisseria meningitidis*	ATCC 13091	SAMN00117532	GCA_000146655.1	Scaffold	2010-08-20
33	*Neisseria gonorrhoeae*	ATCC 49226	SAMN13008815	GCA_016803895.1	Chromosome	2021-02-03
34	*Neisseria gonorrhoeae*	NCTC13798	SAMEA4076741	GCA_900186875.1	Complete	2017-08-15
35	*Neisseria elongate*	ATCC 29315	SAMN02797820	GCA_000818035.1	Complete	2015-01-14
36	*Neisseria elongate*	NCTC11050	SAMEA53328418	GCA_900475815.1	Complete	2018-06-17
37	*Neisseria sicca*	ATCC 29256	SAMN00008842	GCA_000174655.1	Contig	2009-04-24
38	*Neisseria lactamica*	NCTC10617	SAMEA3867448	GCA_901482445.1	Complete	2019-05-11
39	*Neisseria lactamica*	ATCC 23970	SAMN02870721	GCA_000741965.1	Scaffold	2014-08-19
40	*Neisseria polysaccharea*	ATCC 43768	SAMN00008844	GCA_000176735.1	Contig	2009-11-09
41	*Neisseria mucosa*	NCTC 10774	SAMEA104062571	GCA_900454435.1	Contig	2018-07-30
42	*Neisseria mucosa*	ATCC 19696	SAMN08770273	GCA_003028315.1	Complete	2018-04-01
43	*Neisseria weaveri*	NCTC12742	SAMEA104338429	GCA_900638685.1	Complete	2018-12-19
44	*Neisseria weaveri*	ATCC 51223	SAMN02470917	GCA_000224275.2	Contig	2011-08-22
45	*Neisseria cinerea*	NCTC10294	SAMEA3714889	GCA_900475315.1	Complete	2018-06-17
46	*Neisseria cinerea*	ATCC 14685	SAMN00008837	GCA_000173895.1	Contig	2009-02-04
47	*Neisseria subflava*	ATCC 49275	SAMN11461056	GCA_005221305.1	Complete	2019-05-07
48	*Neisseria flavescens*	ATCC 13120	SAMN11461057	GCA_005221285.1	Complete	2019-05-07
49	*Neisseria flavescens*	NCTC8263	SAMEA3936792	GCA_900453805.1	Contig	2018-07-30
50	*Neisseria bacilliformis*	ATCC BAA-1200	SAMN00253995	GCA_000194925.1	Scaffold	2011-04-04
51	*Neisseria animalis*	ATCC 49930	SAMN09853482	GCA_008806995.1	Complete	2019-10-03
52	*Neisseria animalis*	NCTC10212	SAMEA3643316	GCA_900636515.1	Complete	2018-12-19
53	*Neisseria animaloris*	NCTC12227	SAMEA104210758	GCA_900638345.1	Complete	2018-12-19
54	*Neisseria dentiae*	NCTC13012	SAMEA4475693	GCA_900453685.1	Contig	2018-07-30
55	*Neisseria zoodegmatis*	NCTC12230	SAMEA4504057	GCA_900187305.1	Complete	2017-08-15
56	*Neisseria canis*	NCTC10296	SAMEA3871785	GCA_900636765.1	Complete	2018-12-19
57	*Neisseria canis*	ATCC 14687	SAMN06212706	GCA_002108495.1	Contig	2017-04-24

### Identification of Strain SAMN18451419

#### Sampling and Clinical Data

A Gram-negative coccus was isolated from the blood culture of a patient with an intermittent fever that had persisted for 3 weeks. Clinical data were retrieved from the patient’s medical record.

#### Bacterial Isolation and Clinical Identification

The isolate was plated on blood and chocolate agar (Autobio, China) plates at 35°C under 5% (v/v) CO_2_ before testing (Humphries et al., 2021). Single colonies were selected from the plates. For phenotypic characterization, 3% (v/v) hydrogen peroxide and tetramethyl-p-phenylenediamine hydrochloride were used in the catalase and oxidation assays. For molecular characterization, the isolate was submitted to matrix-assisted laser desorption/ionization time-of-flight mass spectrometry (MALDI-TOF MS) using a VITEK MS platform (bioMérieux) and VITEK 2 Compact software (bioMérieux).

#### Whole-Genome Sequencing and Analysis

The *Neisseria* strain described earlier was subjected to WGS. Genomic DNA was extracted using a commercial kit (QIAGEN Gentra Puregene Yeast/Bact Kit, Germany) in accordance with the manufacturer’s instructions. The library for single-molecule real-time (SMRT) sequencing was constructed (insert size 10 kb) using the SMRT Bell Template kit version 1.0; a next-generation sequencing library was generated using the NEBNext Ultra DNA Library Prep Kit (from Illumina), in accordance with the manufacturer’s recommendations. WGSs were obtained using the PacBio Sequel and Illumina NovaSeq PE150 platforms at Beijing Novogene Bioinformatics Technology Co., Ltd. Clean reads were assembled using SMRT Link ver. 5.1.0 (Supplementary Figure 1).

#### Data Availability

The complete genome data of strain SAMN18451419 have been deposited in GenBank with the accession number CP072524^2^.

#### Identification of Virulence-Associated Genes of Strain SAMN18451419

Virulence genes were identified using the Virulence Factor database^3^.

#### PubMLST Analysis of Strain SAMN18451419

After draft genome assembly, we used multi-locus sequence typing (MLST) for taxonomic identification. The assembled scaffolds were submitted to the *Neisseria* MLST website^4^ (Jolley et al., 2018).

### Reclassification of *N. mucosa* and *N. sicca*

#### Phylogenetic Analysis

To characterize the evolutionary relationships among *Neisseria* isolates, we used a core genome-based phylogenetic tree to identify the *Neisseria* genomes [strain SAMN18451419, previously identified *N. mucosa* (*n* = 13) and *N. sicca* (*n* = 16), and 27 type *Neisseria* genomes] (Table 1). All genomes were annotated using RAST^5^. The core genes in *Neisseria* genomes were identified using RAST and Roary^6^. A maximum-likelihood phylogenetic tree based on the core single-nucleotide polymorphism alignments was generated using MegaX (Kumar et al., 2018). Phylogenetic tree visualizations were produced by the Interactive Tree of Life^7^.

#### Average Nucleotide Identity Blast Analysis

ANIb analysis was performed using pyani^8^. The following genomes were analyzed: strain SAMN18451419, previously identified *N. mucosa* (*n* = 13) and *N. sicca* (*n* = 16), and 27 type *Neisseria* strains. Pairwise ANIb data for each strain were clustered and visualized using heatmap. All software mentioned earlier were implemented using the default settings.

## Results

### Case Presentation

**FIGURE 1:**
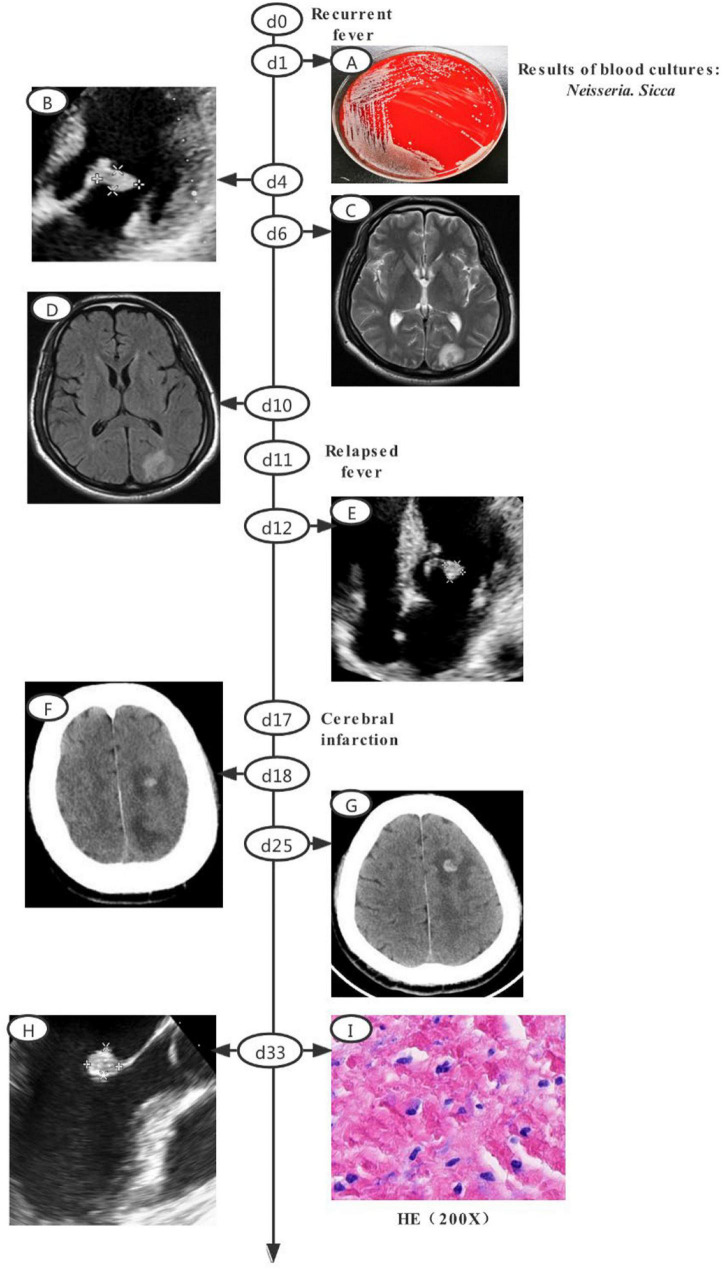
Clinical course of the patient (a schematic). **(A)** Gray-white colonies of strain SAMN18451419 growing on a blood agar plate. **(B)** Echocardiography revealed vegetations attached toe anterior and posterior leaflets of mitral valve, with dimensions of approximately 19 × 10 and 12 × 7.8 mm, respectively. **(C)** Cranial MRI revealed multiple abnormal shadows in left occipital lobe and on both sides of ventricle. **(D)** Cranial contrast-enhanced MRI revealed abnormal enhancement of punctate, patchy, and ring-shaped components of the lesion. **(E)** Echocardiography revealed a post-treatment reduction in vegetation attached to mitral valve. **(F)** CT revealed low-density lesions in left frontal, parietal, and occipital lobes with a small amount of hemorrhage after infarction. **(G)** Cranial CT revealed larger lesions and new low-density lesions in left cerebellar hemisphere. **(H)** Transesophageal echocardiography revealed a highly echoic mass (dimensions of approximately 1.07 × 1.08 × 1.69 cm) attached to anterior mitral valve leaflet. **(I)** HE staining of valvular tissue revealed connective tissue hyperplasia and collagenization, mucus changes, calcification, and both acute and chronic inflammatory cell infiltration and necrosis. MRI, magnetic resonance imaging; CT, computed tomography; HE, Hematoxylin-cosin.

A 52-year-old man with a recurrent fever was admitted (day 0). A schematic of the disease course is shown in Figure 1. Before admission, he had developed non-specific symptoms, including high fever, fatigue, anorexia, nausea, and vomiting (all persisted for 3 weeks). Considering the high leukocyte count (15.9 × 10^9^/L) and C-reactive protein level (144.1 mg/L), despite the absence of an obvious infective focus, empirical outpatient treatment (azlocillin sodium) was administered before admission; this treatment was ineffective. Empirical ticarcillin disodium and clavulanate potassium were prescribed from days 0 to 5. A Gram-negative diplococcus was isolated from blood cultures on day 1. Echocardiography on day 4 revealed vegetations attached to the anterior and posterior leaflets of the mitral valve, with dimensions of approximately 19 × 10 and 12 × 7.8 mm, respectively (Figure 1B). Cranial magnetic resonance imaging (MRI) on day 6 showed multiple abnormal signals in the left occipital lobe and on both sides of the ventricle (Figure 1C). The antibiotics were switched to ceftriaxone and amoxicillin sodium clavulanate potassium, based on the drug sensitivities of the isolate (minimal inhibitory concentration of ceftriaxone ≤0.12 μg/ml; recorded from days 6 to 13). Cranial contrast-enhanced MRI revealed abnormal enhancement of punctate, patchy, and ring-shaped lesional regions (compared with the day 6 MRI image); infectious lesions were first considered on day 10 (Figure 1D). The body temperature returned to the normal level, followed by downward trends in the indicators of inflammation from days 1 to 10. However, a relapse developed on day 11. Follow-up heart ultrasonography showed that the vegetations attached to the mitral valve had shrunken (Figure 1E).

The patient was then transferred to our main campus for further treatment. Piperacillin sodium and tazobactam sodium were prescribed from days 13 to 16. However, the high fever persisted. Follow-up ultrasonography of the heart, as well as cranial MRI, yielded results similar to the findings on day 15. The antibiotics were changed to ceftriaxone sodium and compound sulfamethoxazole from days 16 to 38. Symptoms of cerebral infarction manifested on day 17. Subsequent blood cultures were negative. Cranial contrast-enhanced computed tomography (CT) revealed low-density focus in the left frontal, parietal, and occipital lobes, with minor hemorrhage after infarction on day 18 (Figure 1F). On day 25, cranial CT showed that the lesion had expanded, and new low-density lesions had developed in the left cerebellar hemisphere. The focus of ischemic infarction was compared with the focus on day 18 (Figure 1G). On day 33, mitral valve replacement, tricuspid valvuloplasty, and left atrium folding were performed after preoperative transesophageal echocardiography had revealed a highly echoic mass (dimensions of approximately 1.07 × 1.08 × 1.69 cm) attached to the anterior mitral valve leaflet (Figure 1H). Histology revealed valvular connective tissue hyperplasia and collagenization, mucus changes, calcification, and both acute and chronic inflammatory cell infiltration and necrosis (Figure 1I). Follow-up ultrasonography of the heart (after the operation) revealed normal mechanical valve function without any obvious paravalvular leakage. On day 39, follow-up cranial CT showed that the low-density focus had been absorbed; the size was reduced relative to the size on day 31. The patient was discharged to home on anticoagulant therapy and scheduled for follow-up outpatient review 1 month later.

### Clinical Phenotypic Identification of Strain SAMN18451419

The blood and chocolate agar plates both grew off-white Gram-negative diplococci (Figure 1A). The isolates exhibited both catalase and oxidase. Isolates from both plates were identified as *N. sicca* by VITEK MS and VITEK 2 Compact with 99.9% confidence.

### General Genomic Features of Strain SAMN18451419

Sequencing of strain SAMN18451419 revealed a genome size of 2,566,407 bp with a G+C content of 51.1%. There was one scaffold featuring 2,295 protein-encoding genes, 61 transfer RNAs, and 12 ribosomal RNAs (Supplementary Figure 2).

### Identification of Virulence-Associated Genes in Strain SAMN18451419

The genome encoded 21 putative virulence factors (Supplementary Figure 2).

### PubMLST Analysis of Strain SAMN18451419

Strain SAMN18451419 was identified as a new sequence type using the *Neisseria* public databases for molecular typing and microbial genome diversity (PubMLST)^9^.

### Phylogenetic Analysis of *N. mucosa* and *N. sicca*

**FIGURE 2:**
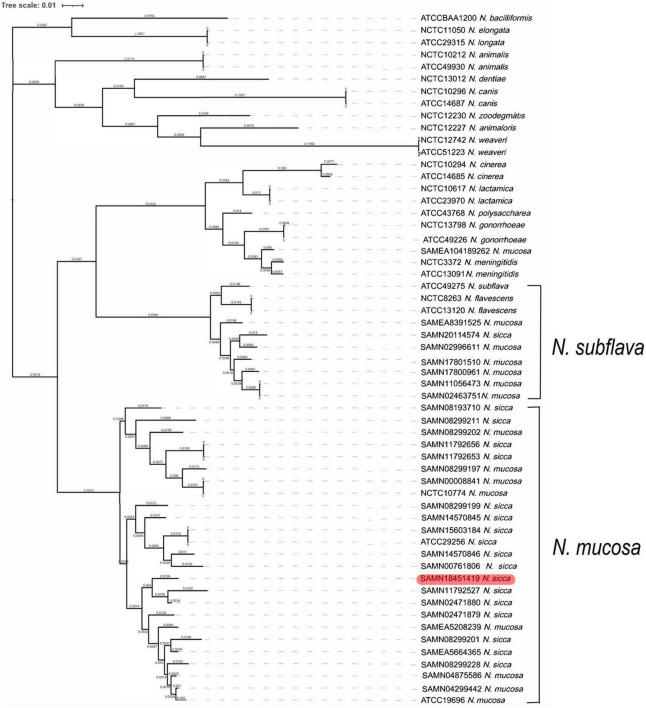
Phylogenetic analysis of 57 *Neisseria* strains. The maximum likelihood tree showed phylogeny of genus *Neisseria* based on WGS. The phylogenetic tree was generated for clinical isolate SAMN18451419, as well as 13 *N. mucosa* strains, 16 *N. sicca* strains, and 27 *Neisseria* type strains (including two *N. mucosa* and one *N. sicca* type strains). Red color code refers to clinical isolate SAMN18451419. WGS, whole-genome sequencing.

We explored the phylogenetic relationships among *N. mucosa*, *N. sicca*, and the 27 *Neisseria* type strains based on the core genomes obtained *via* WGS. Clear separation of the strains listed in Table 1 was evident in the phylogenetic tree (Figure 2). Eight strains originally identified as *N. mucosa* (including two *N. mucosa* type strains ATCC 19696 and NCTC 10774) and 17 strains originally identified as *N. sicca* (including the clinical isolate SAMN18451419 and one *N. sicca* type strain ATCC 29256) clustered into the *N. mucosa* group. Six strains originally identified as *N. mucosa* and one strain originally identified as *N. sicca* clustered into the *N. subflava* group. Notably, strain SAMEA104189262, originally classified as *N. mucosa*, clustered with the *N. meningitidis*-type strains NCTC 3372 and ATCC 13091.

### Average Nucleotide Identity Blast Analysis of *N. mucosa* and *N. sicca*

**FIGURE 3:**
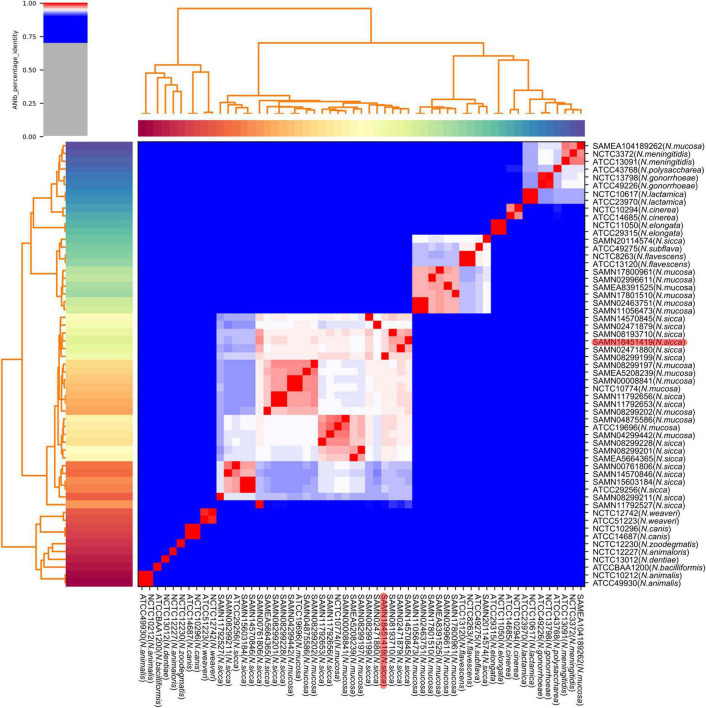
Heatmap and dendrogram of ANIb values of 57 *Neisseria* genomes. Sequences of clinical isolate SAMN18451419, as well as 13 *N. mucosa* and 16 *N. sicca* strains downloaded from GenBank, were characterized by pairwise ANIb with 27 *Neisseria* type strains (including two *N. mucosa* and one *N. sicca* type strains). Red color code refers to clinical isolate SAMN18451419. ANIb, average nucleotide identity blast.

Consistent with the results of phylogenetic analysis, the eight strains originally identified as *N. mucosa* (including two *N. mucosa* type strains ATCC 19696 and NCTC 10774) and the 17 strains originally identified as *N. sicca* (including the clinical isolate SAMN18451419 and one *N. sicca* type strain ATCC 29256) co-clustered. Six strains originally identified as *N. mucosa* and one strain originally identified as *N. sicca* clustered with the *N. subflava* type strain ATCC 49275. One strain originally identified as *N. mucosa* (SAMEA104189262) clustered with two *N. meningitidis* type strains NCTC 3372 and ATCC 13091 (Figure 3). According to the combination of robust phylogenetic and ANIb analysis, the proposed reclassification of previously identified *N. mucosa* and *N. sicca* strains were listed in Table 2.

**TABLE 2:** Summary of proposed reclassification of *N. mucosa* and *N. sicca* strains.

No.	GenBank strain BioSample	Classification	
		Current	Proposed
1	SAMEA8391525	*N. mucosa*	*N. subflava*
2	SAMEA5208239	*N. mucosa*	*N. mucosa*
3	SAMEA104189262	*N. mucosa*	*N. meningitidis*
4	SAMN00008841	*N. mucosa*	*N. mucosa*
5	SAMN02463751	*N. mucosa*	*N. subflava*
6	SAMN02996611	*N. mucosa*	*N. subflava*
7	SAMN04299442	*N. mucosa*	*N. mucosa*
8	SAMN04875586	*N. mucosa*	*N. mucosa*
9	SAMN08299197	*N. mucosa*	*N. mucosa*
10	SAMN08299202	*N. mucosa*	*N. mucosa*
11	SAMN11056473	*N. mucosa*	*N. subflava*
12	SAMN17800961	*N. mucosa*	*N. subflava*
13	SAMN17801510	*N. mucosa*	*N. subflava*
14	SAMN00761806	*N. sicca*	*N. mucosa*
15	SAMEA5664365	*N. sicca*	*N. mucosa*
16	SAMN02471879	*N. sicca*	*N. mucosa*
17	SAMN02471880	*N. sicca*	*N. mucosa*
18	SAMN08193710	*N. sicca*	*N. mucosa*
19	SAMN08299201	*N. sicca*	*N. mucosa*
20	SAMN08299211	*N. sicca*	*N. mucosa*
21	SAMN08299228	*N. sicca*	*N. mucosa*
22	SAMN11792527	*N. sicca*	*N. mucosa*
23	SAMN11792653	*N. sicca*	*N. mucosa*
24	SAMN11792656	*N. sicca*	*N. mucosa*
25	SAMN14570845	*N. sicca*	*N. mucosa*
26	SAMN14570846	*N. sicca*	*N. mucosa*
27	SAMN15603184	*N. sicca*	*N. mucosa*
28	SAMN20114574	*N. sicca*	*N. subflava*
29	SAMN08299199	*N. sicca*	*N. mucosa*
30	SAMN18451419	*N. sicca*	*N. mucosa*
31	NCTC 10774	*N. mucosa*	*N. mucosa*
32	ATCC 10617	*N. mucosa*	*N. mucosa*
33	ATCC 29256	*N. sicca*	*N. mucosa*

## Discussion

*N. mucosa* and *N. sicca* are genetically closely related and have a contentious taxonomic history [described in Bergey’s Manual of Systematic Bacteriology and addressed by Bennett et al. (2012)]. Traditional bacterial taxonomic assignments use phenotypic approaches; these are generally unsatisfactory because phenotypes can vary (Brodie et al., 1971). WGS has greatly assisted bacterial taxonomy. Data ranging from small regions (from which 16S ribosomal RNAs are transcribed) to the entire genome are readily available (Mechergui et al., 2014). Modern genomic analysis approaches based on WGS, rMLST, cgMLST, ANIb, and phylogenetic analysis have flourished recently (Bennett et al., 2012; Zong, 2020).

We used phylogenetic analysis to reclassify the originally (mis)identified *N. sicca* and *N. mucosa* strains into the *N. mucosa* group, confirming the findings by Bennett et al. (2012). The term “*N. mucosa* group” is used because *N. mucosa* [originally termed *Diplococcus mucosus* by Von Lingelsheim in 1906 (Bennett et al., 2012)] was the first such species to be identified (Tønjum, 2005). The *N. subflava* group, in which one *N. sicca*, six *N. mucosa*, one *N. subflava*, and two *N. flavescens* strains co-clustered in our results, was also named in accordance with the same principle in the work by Bennett et al. (2012). Only one strain originally identified as *N. sicca* belonged in the *N. subflava* group in the work by Bennett et al. (2012), whereas we found that one strain originally identified as *N. sicca* and six strains originally identified as *N. mucosa* clustered into that group. The reason may be that six *N. mucosa* strains, most of which sequences were uploaded to GenBank after 2012, analyzed in our research, were not enrolled by Bennett et al. (2012). According to our results, some originally misidentified *N. mucosa* and *N. sicca* species seem as variants of *N. subflava.* The *N. mucosa* and *N. subflava* groups of the phylogenetic tree also clustered in the ANIb heatmap, which revealed ANIb analysis verified our initial conclusion.

The genus *Neisseria* includes the two pathogenic species, *N. meningitidis* and *N. gonorrhoeae*; the remaining species are opportunistic pathogens (Caugant and Brynildsrud, 2020). Compared with the opportunistic pathogen *Neisseria* species, *N. meningitidis* causes significant morbidity and mortality (Wang et al., 2019). Notably, one strain originally identified as *N. mucosa* was reclassified as *N. meningitidis.* It reveals some GenBank genomes are clearly not curated or checked, creating taxonomic errors. We found that strain SAMN18451419 from a patient with congenital heart disease was misidentified by VITEK MS as *N. sicca*. It indicates that both MS and GenBank databases require revision of *Neisseria* classification.

## Conclusion

We offered a detailed and well-supported description of the phylogenetic relationship between the (erroneously) originally classified *N. mucosa* and *N. sicca* strains, as confirmed by ANIb analysis. The original *N. mucosa* and *N. sicca* strains were reclassified into the *N. mucosa* group and *N. subflava* group *via* high-resolution genomic taxonomy. The GenBank and MALDI-TOF MS databases thus require correction. In addition, the classification strategy for originally identified *N. mucosa* and *N. sicca* strains in GenBank is supposed to be updated with the progressive development of genomic analysis approaches.

## Data Availability Statement

The datasets presented in this study can be found in online repositories. The names of the repositories and accession numbers can be found in the article/Supplementary Material.

## Ethics Statement

The study was conducted in accordance with the Declaration of Helsinki and was approved [2021IIT026 (fast track)] by the Clinical Research Ethics Committee of the First Affiliated Hospital, Zhejiang University School of Medicine. The patient provided the written informed consent to participate in this study. Written informed consent was obtained from the individual for the publication of any potentially identifiable images or data included in this article.

## Author Contributions

YZ and BZ designed the research. YJ, HX, YZ, and BZ analyzed and collected data, and wrote the manuscript. QY, BG, ZW, TW, YL, and XY collected the clinical data. All authors read and approved the final manuscript.

## Conflict of Interest

The authors declare that the research was conducted in the absence of any commercial or financial relationships that could be construed as a potential conflict of interest.

## Publisher’s Note

All claims expressed in this article are solely those of the authors and do not necessarily represent those of their affiliated organizations, or those of the publisher, the editors and the reviewers. Any product that may be evaluated in this article, or claim that may be made by its manufacturer, is not guaranteed or endorsed by the publisher.

## Footnotes

1
https://www.ncbi.nlm.nih.gov/assembly/?term=Neisseria

2
https://www.ncbi.nlm.nih.gov/nuccore/CP072524.1/

3
http://www.mgc.ac.cn/VFs/main.htm

4
https://pubmlst.org/neisseria/

5
https://rast.Nmpdr.org

6
https://github.com/deminu/Roary

7
https://itol.embl.de/

8
https://github.com/widdowquinn/pyani

9
https://pubmlst.org/bigsdb?db=pubmlst_neisseria_seqdef&page=sequenceQuery
