# Supplementary material for: Confirmation of the Need for Reclassification of Neisseria mucosa and Neisseria sicca Using Average Nucleotide Identity Blast and Phylogenetic Analysis of Whole-Genome Sequencing: Hinted by Clinical Misclassification of a Neisseria mucosa Strain
Source: Front Microbiol. 2022 Feb 21;12:780183. doi: 10.3389/fmicb.2021.780183 (PMC8909641; doi:10.3389/fmicb.2021.780183)
Supplement: Supplementary file 1 [file Data_Sheet_1.docx]

Supplementary Material

**Supplementary Figure 1 Whole genome sequencing and analysis process of reclassification.**


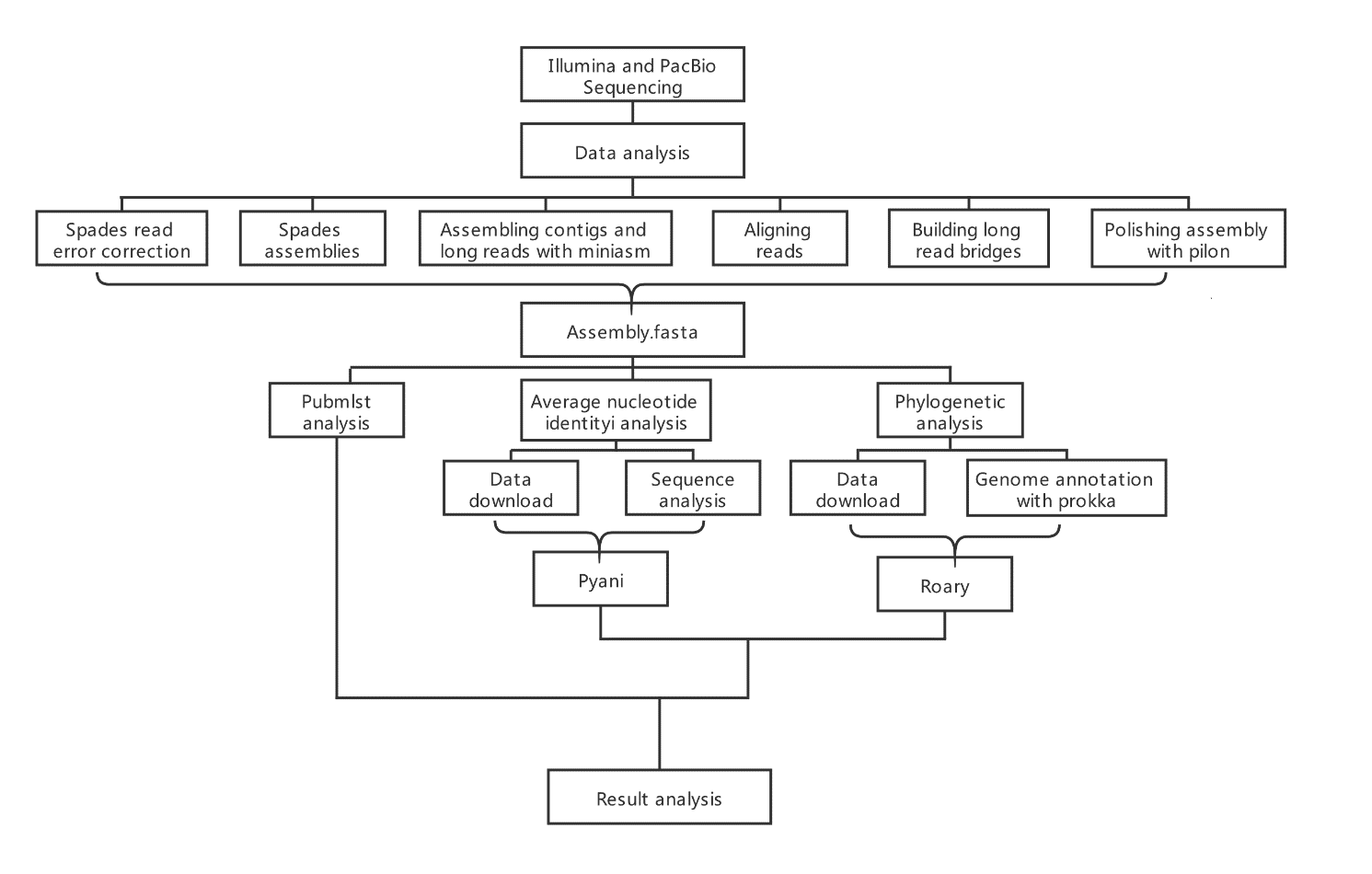


**Supplementary Figure 2 Schematic representation of general genome features of strain SAMN18451419**

**
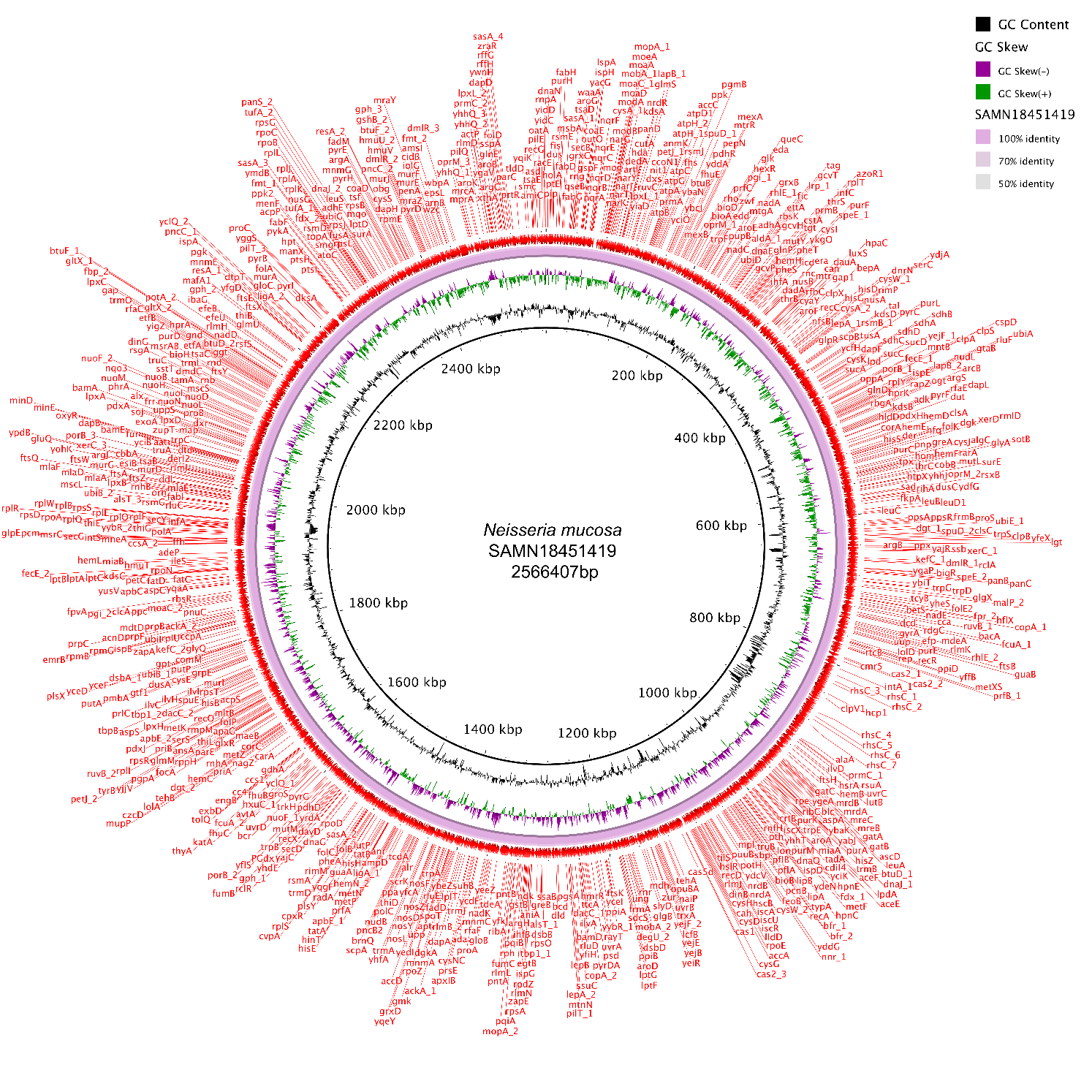
**
